# Stimulus dependent diversity and stereotypy in the output of an olfactory functional unit

**DOI:** 10.1038/s41467-018-03837-1

**Published:** 2018-04-09

**Authors:** Ezequiel M. Arneodo, Kristina B. Penikis, Neil Rabinowitz, Angela Licata, Annika Cichy, Jingji Zhang, Thomas Bozza, Dmitry Rinberg

**Affiliations:** 1 Neuroscience Institute, NYU Langone Health, 435 E 30h St, New York, NY 10016 USA; 2 Center for Neural Science, New York University, 4 Washington Place, New York, NY 10003 USA; 3 Howard Hughes Medical Institute, New York, 10003 NY USA; 4 Department of Neurobiology, Northwestern University, 2205 Tech Drive, Evanston, IL 60208 USA; 5 Present Address: Biocircuits Institute, University of California San Diego, 9500 Gilman Dr, La Jolla, CA USA; 6 DeepMind, 6 Pancras Square, London, N1C 4AG UK

## Abstract

Olfactory inputs are organized in an array of functional units (glomeruli), each relaying information from sensory neurons expressing a given odorant receptor to a small population of output neurons, mitral/tufted (MT) cells. MT cells respond heterogeneously to odorants, and how the responses encode stimulus features is unknown. We recorded in awake mice responses from “sister” MT cells that receive input from a functionally characterized, genetically identified glomerulus, corresponding to a specific receptor (M72). Despite receiving similar inputs, sister MT cells exhibit temporally diverse, concentration-dependent, excitatory and inhibitory responses to most M72 ligands. In contrast, the strongest known ligand for M72 elicits temporally stereotyped, early excitatory responses in sister MT cells, consistent across a range of concentrations. Our data suggest that information about ligand affinity is encoded in the collective stereotypy or diversity of activity among sister MT cells within a glomerular functional unit in a concentration-tolerant manner.

## Abstract (web summary)

Mitral/tufted (MT) cells connect to a single glomerulus and receive inputs from sensory neurons expressing the same odorant receptor. Here the authors report that sister MT cells connected to the M72 glomerulus exhibit variable responses to most M72 ligands but respond in a reproducible and stereotyped way to a high-affinity M72 ligand.

## Introduction

Objects in the world are represented by complex patterns of activity in peripheral sensory neurons. Prior to reaching cortical areas, these representations are transformed and reformatted. One of the central challenges in sensory neuroscience is to understand the functional role and computational logic of these transformations in extracting salient information about the environment.

In mammals, the olfactory bulb is the single interface between primary olfactory sensory neurons (OSNs) and higher brain regions such as piriform cortex. OSNs carry information about odors to the olfactory bulb via a vast array of glomeruli. Each glomerulus is a functional unit, collecting input from OSNs that express a single olfactory receptor gene^1^ and that share similar response properties^2^. Each glomerulus provides exclusive excitatory input to a set of 10–20 mitral/tufted (MT) cells, which project to higher brain areas^3^. The output of a given MT cell depends not only on the response of the glomerulus providing its input but also on the activity of the complex network of inhibitory interneurons within which it is embedded^3^.

It is still not understood how odor information is represented by MT cells. As an odor is inhaled, a unique subset of glomeruli is activated, resulting in a spatiotemporal pattern that evolves over the course of the respiration cycle^4,5^. Once this input reaches the MT layer, however, there is substantial heterogeneity among cellular responses. The population of MT cells responds to a given odor with various combinations of temporally patterned excitation and inhibition^6,7^. Recent observations from anesthetized animals suggest that MT cells that are connected to the same glomerulus (sister MT cells) respond to odors with variable excitation, inhibition, and response timing^8–10^. However, it is not clear how the complexity and diversity of MT responses relate to specific attributes of the odor stimulus. What determines whether sister MT cells show uniform or divergent responses to a given odorant? Are these response properties stable under natural variation in the odor signal, such as changes to odor concentration? Given that sister MT cells do not always behave in a unified way, what information can this subpopulation of cells convey about an odor?

Here we provide an answer to these questions by assessing the odor representation at the input and output of a glomerular functional unit in awake mice. Using a combination of mouse genetics, electrophysiology, and imaging, we define the functional properties of inputs to a genetically tagged glomerulus, and then use optogenetics to identify MT cells that get input from this glomerulus. We observe, for the first time, stimulus-dependent diversity or stereotypy among sister MT cell responses in awake animals. We find that relative ligand affinity for a given odorant receptor is a major determinant of whether the MT cells respond in a uniform manner, and whether individual cell responses are consistent across concentrations. Our results directly link a fundamental stimulus property with a robust, concentration-invariant response feature, and suggest a novel way of looking at olfactory coding.

## Results

### Inputs and outputs of the M72 glomerulus

To study how a single channel in the olfactory bulb, an ensemble of MT cells connected to the same glomerulus, processes stimulus information, we characterized the inputs and outputs of the mouse M72 glomerulus.

**Fig. 1:**
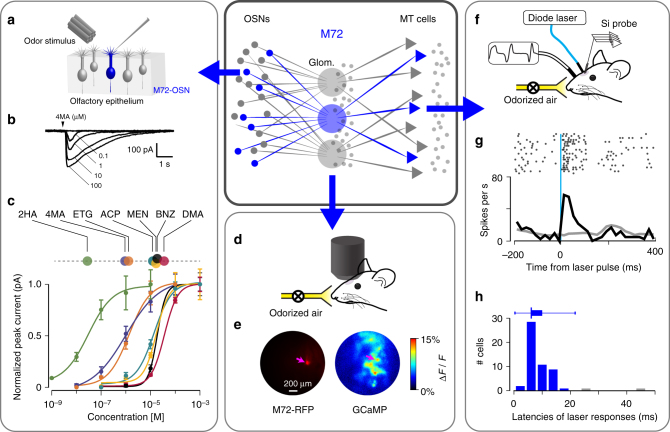
Characterizing information in a single channel of the mouse olfactory bulb. Central insert: schematic of the olfactory bulb network. Axons from OSNs expressing the same receptor gene converge to form glomeruli, each providing the sole excitatory input to a few MT cells. Odor signals are subject to significant modification by a network of inhibitory neurons (small gray dots). **a** Experimental setup for characterizing OSN responses to odor. Patch clamp recordings are made from dendrites of fluorescently labeled OSNs expressing the M72 receptor. **b** Example traces of OSN odor responses. **c** Normalized dose–response curves for seven M72 ligands fitted by the Hill equation (*n* = 5–7 OSNs per odorant; mean ± SEM); EC_50_ values indicated in linear plot above. Odors used: 2-hydroxyacetophenone (2HA); ethyl tiglate (ETG); 4-methyl acetophenone (4MA); acetophenone (ACP); menthone (MEN); benzaldehyde (BNZ); and 2,4-dimethyl acetophenone (DMA). EC_50_ values are given in Supplementary Table 1. **d** Experimental setup for imaging. An awake, head-fixed mouse (OMP-GCaMP + M72-RFP) with implanted window above the OB is positioned under the microscope. **e** Left: image of a RFP M72 glomerulus. Right: Ca^2+^ image of glomerular response to an odor (2HA). M72 glomerulus here and further is marked by magenta arrow. **f** Experimental setup for in vivo recording of odor responses from MT cells connected to the M72 glomerulus. A head-fixed mouse is positioned in front of the odor port. The sniff signal is recorded by a pressure sensor via a cannula implanted in the nasal cavity. Brief pulses of blue light are delivered to the ChR2-expressing M72 glomerulus through an optical fiber positioned above the glomerulus. MT cell responses are recorded with a Si-probe inserted nearby. **g** Example of MT cell excitation following laser stimulation of the M72 glomerulus. Raster plot (upper panel) and PSTH (lower panel) around the onset of a 1 ms pulse showing the stimulus response (black line) and the baseline activity (gray line). **h** Distribution of response latencies to a 1 ms, 5–10 mW light pulse. Light-responsive cells with latencies longer than 20 ms (colored gray in the histogram) were excluded from the analysis

First, to characterize the input, we measured the responses of genetically identified M72-expressing OSNs (M72-OSNs) to a defined set of M72 ligands in a semi-intact preparation of the olfactory epithelium^11^. The dendritic knobs of fluorescently labeled OSNs from M72-GFP mice^12^ were targeted for recording via perforated patch (Fig. 1a,b). The relative sensitivities of M72-OSNs to each ligand covered a large range of receptor sensitivities: concentration at half-maximal response (EC_50_) values of the seven odorants spanned three orders of magnitude, from 0.03 to 36 µM (Fig. 1c, Supplementary Table 1). In all figures, we present odors rank-ordered by the M72-OSN sensitivity, from least sensitive (high EC_50_) on the left to most sensitive (low EC_50_) on the right.

Second, to confirm that the M72 ligands would drive MT cells in vivo, we imaged presynaptic OSN activity in identified M72 glomeruli in awake mice (Fig. 1d, e). We used a strain of mice in which all the OSNs express the calcium activity indicator GCaMP3, and in which M72-OSNs also express the red fluorescent protein (RFP). This allowed us to assess the level of activation of M72 (and surrounding) glomeruli for each of the odorant stimuli and concentrations used to record MT cell activity.

Third, to characterize the output of the M72 glomerulus, we measured responses of M72-MT cells to the same odorants. To do so, we developed a novel method to identify these cells in awake, freely breathing animals (Fig. 1f). We used a strain of mice in which M72-OSNs express a channelrhodopsin2-yellow fluorescent protein fusion protein (ChR2-YFP) and are therefore light-sensitive^13^. We periodically stimulated the M72 glomerulus with a 473 nm light pulse while recording extracellular activity in the olfactory bulb (Fig. 1f). Those cells whose firing rate increased shortly after light stimulation were considered putative M72-MT cells (Fig. 1g). The distribution of light-evoked response latencies had its mode and median at 6 ms (Fig. 1h, see Methods); we excluded cells with latencies slower than 20 ms as likely being more than one synapse from the M72 glomerulus. Most of these putative M72-MT cells were recorded from different animals. To compare M72-MT cells with the general MT population, we also recorded generic (i.e., non-M72) cells. No differences were evident between M72-MT and generic MT cell populations in the distributions of spontaneous firing rate, preferred sniff phase, and recording depth (Supplementary Fig. 1). In total, we recorded *N* = 53 M72-MT cells and 312 generic MT cells.

### Functional characterization of MT cells

**Fig. 2:**
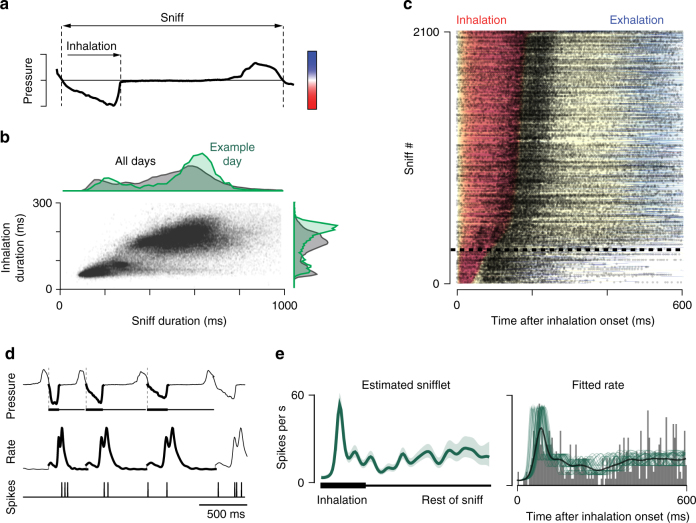
MT cell activity depends on sniff dynamics. **a** A pressure signal showing one complete sniff cycle, recorded from the mouse nasal cavity. **b** Scatter plot of all inhalation and sniff durations, collated across mice. Top: marginal histogram of sniff durations, across all sessions and mice (black), and from one example session (green). Right: marginal histogram of inhalation durations. **c** Spiking of MT cells depends on inhalation duration. Black dots: raster of spike times of an example M72-MT cell across 2100 inhalations, during baseline (no odor) condition. Responses are aligned to the onset of inhalation, and are sorted (rows) by inhalation duration. Colored background shows sniff phase: red = inhalation; blue = exhalation. The color map is positioned in **a**, aligned with the pressure axis. Horizontal dashed line demarcates the rarer fast inhalations (below) from the more common slower inhalations (above). **d** Snifflet model of MT cell responses. Top: pressure traces of three successive sniffs of short, medium, and longer inhalation duration. Inhalation periods illustrated with thickened lines. Middle: a model fit of the sniff-induced firing rate of the MT cell following a particular temporal pattern, denoted as a “snifflet”. Bottom: the observed spikes. The time courses of the snifflets are the free parameters of the model; these are fitted to each cell, for each stimulus condition, given the observed spikes. **e** Snifflet fit to an example M72-MT cell response to a single odor. Left: estimated snifflet for this cell/odor. Shaded region: ±1 SEM. Here and further: the time axis for a snifflet is shown as a thick bar corresponding to the normalized duration of inhalation, followed by a thin bar for the rest of the normalized sniff. Right: gray bars show the trial-averaged PSTH; thick black line shows average firing rate across trials; thin green lines show the model-fitted firing rates on each trial, given the dilations induced by different inhalations

MT cell activity is strongly influenced by the temporal dynamics of respiration^6,14^ and the duration of odor exposure. In freely breathing, head-fixed mice, there is considerable variability in sniff frequency and duration (Fig. 2a, b, c). Such variability causes peri-stimulus time histograms (PSTHs) of MT cell odor responses to be temporally smeared (Fig. 2c)^6^, and makes it difficult to compare MT responses between different mice with different sniff patterns. Here we monitored MT responses in relation to the sniff cycle and focused our analyses on slower sniffs—those with an inhalation duration > 100 ms (Fig. 2c)—because they comprised 75% of all sniffs across all mice, while the rarer, fast sniffs seemed to mark a distinct behavioral state (Supplementary Fig. 2). MT responses during fast sniffs showed the same general trends as during slow sniffs, but the lower number of events precluded a rigorous analysis (Supplementary Fig. 3). Finally, to avoid adaptation effects, we restricted our analyses to the first sniff cycle after odor onset.

To further account for variability due to sniff dynamics, we developed a statistical model for the responses of MT cells that factored in both the dependency on the stimulus and on the pattern of sniffing. We modeled the spiking response of an MT cell as arising due to an odor-dependent firing rate pattern, a “snifflet”, that gets temporally dilated as a function of the duration of each sniff (Fig. 2d). The model fits best when the temporal dilation is a function of the inhalation duration (Supplementary Fig. 4). To characterize how each cell responds to each odor, we estimate the corresponding snifflet from the observed spiking data (Fig. 2e), which we accomplish using fast Bayesian methods^15,16^. This snifflet representation factored out the variability in sniff duration, allowing us to compare activity across cells and across mice.

From this point forward, we characterize the odor-evoked MT cell responses by comparing the corresponding snifflets. Our results, however, do not depend on the specifics of these modeling decisions: when we used the snifflet model without temporal dilation, the results were qualitatively identical (Supplementary Fig. 5).

### Diversity and stereotypy of MT cell responses

**Fig. 3:**
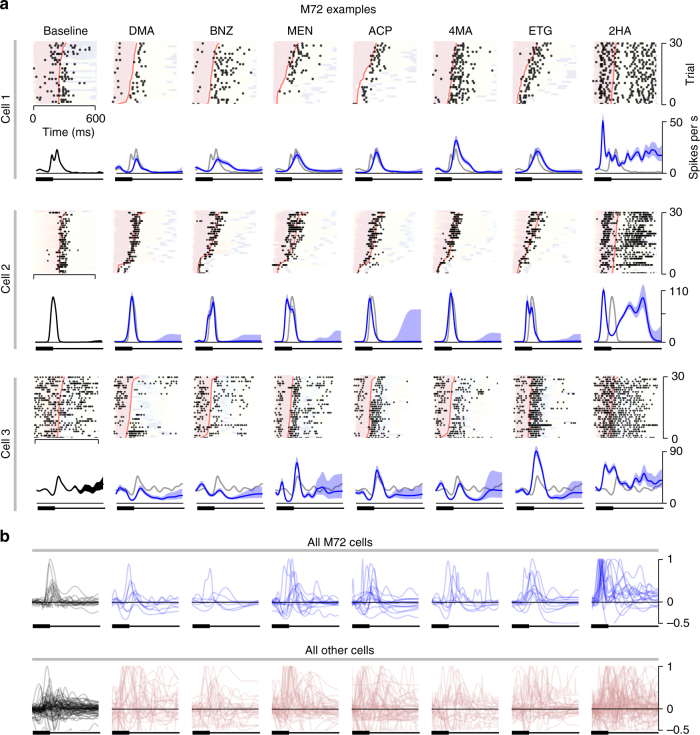
MT cell responses to odor presentation**. a** Raster plots and snifflet estimates for three example M72-MT cells, for each odor (sorted by affinity) and baseline conditions. Rasters are shown in real time, as in Fig. 2, for the first sniff of each trial. Trials are sorted by inhalation duration, background color corresponds to sniff phase (as in Fig. 2), and inhalation offset is marked in red. The corresponding snifflet for each odor and cell (below, blue) was estimated based on the first sniff after odor onset. Baseline snifflets (first column, black) were estimated from activity in the three seconds prior to each odor onset. Baseline snifflets are overlaid in gray in each odor panel for comparison. Time axis for snifflets as in Fig. 2. **b** Top row: estimated snifflets from all recorded M72-MT cells. The snifflet of each cell is normalized such that a cell’s mean snifflet value across odors at inhalation onset is zero, and its maximum peak across odors is unitary. Snifflets are omitted if the cell’s responses were best described as constant over the duration of the sniff. Note the substantial variability in snifflet shapes for all odors except 2HA. Bottom row: all snifflets from the population of non-M72-MT cells

Despite receiving input from functionally similar OSNs, we observed a striking degree of response diversity across M72-MT cells. This diversity was evident directly in the raw patterns of activity, illustrated in the snifflet and raster plots in Fig. 3. Diversity was observed for most M72 ligands, presented at the same approximate concentration, 0.075 ± 0.01 µM. Interestingly, responses to a particular ligand, 2-hydroxyacetophenone (2HA), were less variable across the M72-MT cell population (right column of Fig. 3). Almost every cell responded to this odor with a short-latency increase in firing rate. After this robust, reproducible burst, the responses diverged and exhibited considerable variability. Notably, 2HA is the strongest ligand yet identified for M72, with an EC_50_ that is two orders of magnitude lower than that of any other identified M72 ligand^11^ (Fig. 1c, Supplementary Table 1).

**Fig. 4:**
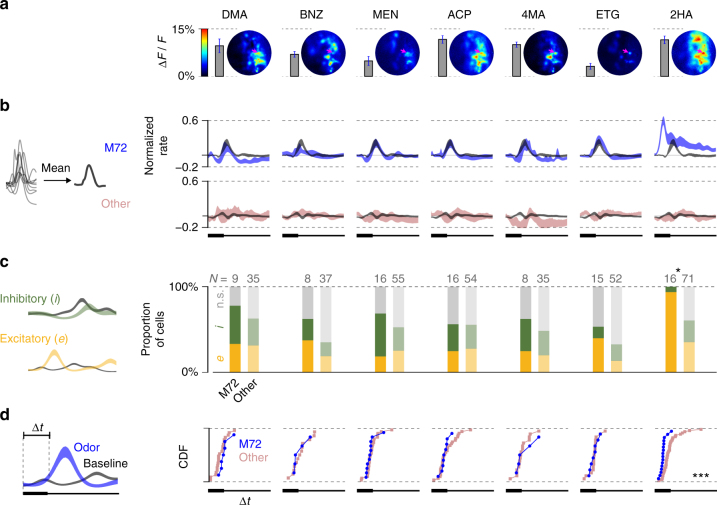
Response variability/stereotypy across the M72-MT population. **a** Map of Ca^2+^ activity of OSN terminals in the vicinity of M72 glomerulus (magenta arrow) for all odors. Vertical bars indicate the average level of M72 glomerulus activation for each odor. **b** Mean snifflet responses. Left (for all following): schematic of measurement. Right: mean normalized snifflets for each odor (as in Fig. 3b), averaged across cells. M72-MT cells in blue; generic MT cells in pink; mean baseline snifflets in gray. Shaded region denotes ±1 SEM. **c** Polarity of the first significant response. Right: histograms of first response polarity (yellow: excitatory; green: inhibitory; gray: no significant rate change) for M72-MT cells (left bar; bold) and generic MT cells (right bar; desaturated). Statistical tests: Pearson’s chi-squared, comparing number of excitatory/inhibitory responses for M72 and generic cells (here and further: *p < 0.05; **p < 0.01; and ***p < 0.001). **d** Latency to first significant response. Significance is measured as a 3*σ* difference between the odor-evoked snifflet and the baseline snifflet. Right: cumulative distribution functions (CDFs) of response latencies among the M72-MT (blue) and generic MT (pink) populations. Cells for which odor-evoked activity did not significantly deviate from baseline are omitted. For all odors except 2HA (last column), there is no significant difference between the latency distributions of the M72-MT population and of the generic MT population (Kolmogorov–Smirnoff two-sample tests)

The differences in M72-MT behavior for 2HA and other ligands cannot be attributed simply to different levels of parent glomerulus activation, since 2HA and other odorants (like 2,4-dimethyl acetophenone, acetophenone, and 4-methyl acetophenone) evoked similar magnitude responses in the M72 glomerulus at the concentrations presented (Fig. 4a). Thus, direct feedforward activation of M72-MT cells via their parent glomerulus does not alone determine whether their responses are diverse or stereotyped.

To quantify the diversity across cell responses, we constructed several metrics (Fig. 4b–d). First, for each odor, we computed the mean response of the ensemble of MT cells. We normalized each cell’s set of snifflets by the snifflet with the largest amplitude and then averaged these across cells. The mean responses of M72-MT cells to most odorants were barely distinguishable from their respective mean responses in the baseline (no odor) condition (Fig. 4b). In contrast, the excitatory response to the strongest ligand (2HA) was still present in the mean activity.

Second, we compared the polarity of the response of each cell to each odor. For each cell and odor, we labeled the response as excitatory or inhibitory, based on the sign of the first significant (3*σ*) deviation of the cell’s odor-evoked snifflet from its baseline (i.e., no odor) snifflet (Fig. 4c, left). For all odors except 2HA, there was considerable diversity amongst M72-MT cells in first response polarity, with roughly one-third of cells having an excitatory response, one-third having an inhibitory response, and the remainder being unresponsive to the odor (i.e., no significant deviation from baseline). These distributions were indistinguishable from those found amongst the generic MT cell population (Fig. 4c, right; Pearson’s chi-squared tests). Again, the exception was 2HA, for which almost every M72-MT cell’s first significant response was excitatory.

Third, we compared the onset latencies of odor responses. We computed these as the time of first significant deviation of a cell’s odor-evoked snifflet from its baseline snifflet (Fig. 4d, left). For all odors but 2HA, the distributions of latencies for M72-MT cells were indistinguishable from those seen amongst the generic MT cell population (Fig. 4d, right; Kolmogorov–Smirnov tests). For 2HA, response latencies amongst M72-MT cells were consistently short (Fig. 4d).

Finally, we found that these properties did not significantly covary with the depth of the recording site (Supplementary Fig. 1a), mean spontaneous firing rate (Supplementary Fig. 1b), or preferred phase of firing during baseline sniffing (Supplementary Fig. 1c). This observation suggests that response differences are not attributable to differences in neuron types (i.e., mitral vs tufted cells).

In summary, although the M72-MT cells receive common input from sensory neurons, their responses to ligands of M72-OSNs are typically as diverse as the rest of the MT population. The exception to this pattern is a high-affinity M72 ligand, 2HA, to which M72-MT cells respond with an initially stereotyped temporal profile, characterized by a strong, short-latency, excitatory transient.

### Population response stereotypy across concentration

The experiment above reveals two different response modes for the M72-MT population: cells can either respond with similar temporal profiles (as we see for 2HA); or with a diverse range of temporal profiles (as we see for all other odors). But which feature of odor stimuli determines the population response mode? Is it the identity of a stimulus (i.e., ligand affinity) or the effective concentration of a stimulus?

To address this question, we selected two odors—menthone (MEN), a weaker ligand, and 2HA, the strongest ligand—and presented them at concentrations spanning two orders of magnitude (*N* = 14–16 M72 and 107–167 generic MT cells; not every cell tested on every odor/concentration condition). With respect to the originally tested concentration, we decreased the concentration of 2HA 10-fold (*C*_−1_) and 100-fold (*C*_−2_), and both increased and decreased the concentration of MEN 10-fold (*C*_+1_ and *C*_−1_, respectively).

**Fig. 5:**
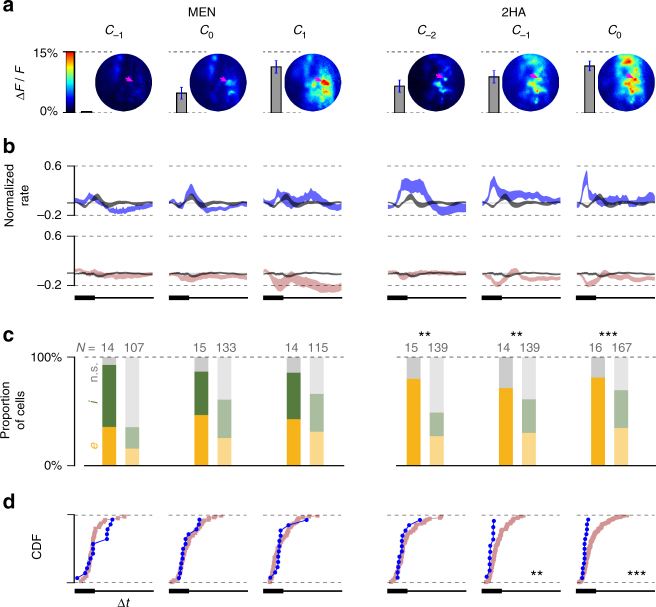
Robustness of population diversity/stereotypy across odor concentrations. Panels as in Fig. 4. **a** Map of Ca^2+^ activity for each odor, and vertical bars indicating the average level of M72 glomerulus activation. Left three columns show increasing concentrations of MEN, a weak ligand for M72-OSNs. Right three columns show increasing concentrations of 2HA, a strong ligand for M72-OSNs. *C*_0_ denotes concentrations equivalent to stimuli presented in Fig. 4; *C*_−2_, *C*_−1_, and *C*_1_ denote concentrations of 0.01*C*_0_, 0.1*C*_0_, and 10*C*_0_, respectively. (Note: due to differences in the experimental setups, in imaging experiments the concentrations for MEN were 1.8× lower than the corresponding concentrations in electrophysiological experiments, and 2× higher for 2HA.) **b** Mean snifflet responses for M72-MT (blue) and other (pink) populations. **c** Distribution of the polarities of the first significant responses for M72 (left bar; bold) and other (right bar; desaturated) MT cells. **d** CDFs of the latency to first significant response

As the concentration of MEN changed, the level of M72 glomerulus activation varied from almost no response at the lowest concentration to a near saturating response at the highest concentration (Fig. 5a). Despite this significant change in glomerular activation, the M72-MT responses remained as diverse as the generic MT responses (Fig. 5b–d, left). In contrast, M72-MT responses to 2HA remained stereotyped at all concentrations (Fig. 5b–d, right). Thus, the diversity of M72-MT cell responses is dependent on odor identity, and not on concentration.

### Single-cell response stereotypy across concentration

Thus far, we have shown that the M72-MT cell population responds in a stereotyped way to a strong ligand, but with considerable diversity to other ligands. This raises the question of whether individual MT cells respond in a different manner to these two classes of stimuli.

**Fig. 6:**
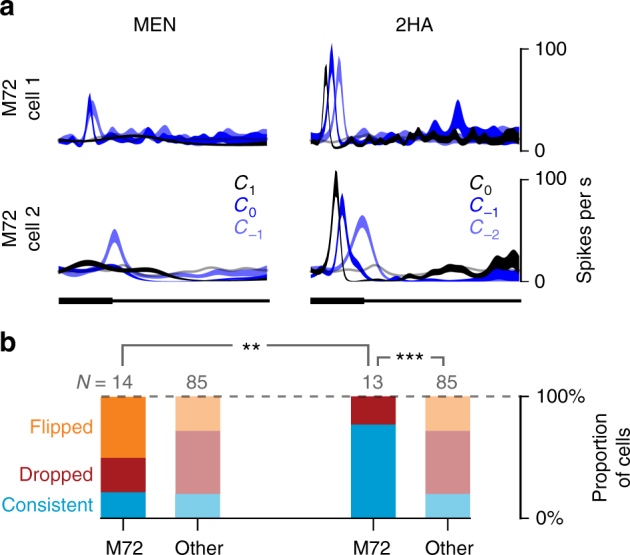
Individual M72-MT cells’ responses are consistent across concentrations for 2HA but not for MEN. **a** Odor responses of two example M72-MT cells to three concentrations of MEN (weak ligand) and 2HA (strong ligand). Shaded areas denote ±1 SEM; light gray trace shows baseline response. **b** Distributions of cells by category of concentration dependency for MEN (left) and 2HA (right). We define three categories: "consistent" (cyan)—the response polarity is the same for all three concentrations; "dropped" (red)—there is at least one concentration at which the response did not significantly differ from baseline; and "flipped" (orange)—the response polarity is different at different concentrations. Cells for which the response was not significantly different from baseline in all three concentrations were omitted. Pairwise comparisons: chi-squared tests

Analyzing individual cells from the second dataset above, we found that changing the concentration of a single odorant could affect single MT cell responses in different ways. As shown in Fig. 6a, the responses of two M72-MT cells to 2HA were consistent at different concentrations: these cells displayed an early excitatory transient at all three concentrations, with the onset latency decreasing as concentration increased, similar to recent reports^17^. Conversely, responses of the same cells to MEN significantly changed with concentration (Fig. 6a, left): increasing the concentration of MEN could attenuate or even reverse an excitatory response observed at a lower concentration.

To quantify these observations, we categorized the concentration dependency of each cell’s response to each odor, based on its first significant deviation from the baseline. We assigned the label "consistent" if the cell’s first significant response to the odor was always excitatory or always inhibitory across concentrations; "dropped", if there was a response for one or two of the concentrations, but no significant response for the other(s); or "flipped", if responses of opposite polarity were observed at different concentrations. We found that the majority of M72-MT cells had consistent initial responses across concentrations of 2HA, while the distribution of response categories was mixed for MEN and for the generic MT population (Fig. 6b).

Thus, a high-affinity ligand not only elicits stereotypic responses across M72-MT cells but also evokes temporal response patterns within cells that are robust to changes in concentration across two orders of magnitude. These observations do not appear to hold for other ligands of M72, nor amongst cells of the general MT population.

## Discussion

Glomeruli are considered functional units in early olfactory processing. Here we have studied for the first time the odor-evoked responses in MT cells that receive input from a genetically identified glomerulus in awake, freely breathing animals. We optogenetically identified sister MT cells that receive excitatory drive from the glomerulus of the M72 odorant receptor, recorded their responses to a set of well-characterized M72 ligands, and analyzed the data using novel statistical tools (Figs. 1 and 2). Despite receiving excitatory drive from functionally similar sensory neurons, M72-MT cells responded to most odorants with highly diverse temporal patterns that were as heterogeneous as those found in the generic MT population. However, this response diversity was not observed in response to 2HA, which has the highest apparent affinity of all identified M72 receptor agonists (Figs. 3 and 4). M72-MT responses to 2HA almost always included a stereotyped early excitatory transient, after which response diversity resumed. These odor-specific patterns of response diversity and stereotypy remained unchanged across odor concentration (Figs. 5 and 6), suggesting that they do not depend on how strongly the glomerulus is activated. Our data indicate that MT cells within a specific olfactory functional unit encode a strong ligand in a markedly different way than weaker ligands.

Previous studies^6,7^ have demonstrated that responses amongst randomly selected MT cells in awake mice are diverse in their polarity and timing across the sniff cycle. Our results suggest that this diversity cannot be attributed solely to different sources of glomerular feedforward input, as we observed that MT cells sharing a common glomerulus show similar response diversity to most odorants.

There are multiple potential sources that could account for the observed MT cell response diversity: (1) variability across animals; (2) variation in intrinsic biophysical properties across cells^18,19^, including differences between mitral and tufted cells^20^; (3) non-homogeneity of excitatory synaptic connections within a glomerulus; and (4) heterogeneity of inhibitory network connectivity. While the first three factors may play some role in the observed diversity of the responses, they cannot easily explain the fact that this diversity vanishes with a strong ligand, nor can they explain that a strong ligand (but not a weak ligand) evokes stereotypical responses over a range of concentrations.

A significant source of between-cell response variability comes from the rich inhibitory network, which includes granule cells, periglomerular, and other inhibitory cells in the olfactory bulb. The connections from granule cells to MT cells are sparse and heterogeneously distributed^3,21,22^, as are the connections from the periglomerular inhibitory network^23,24^. Functionally, too, the activity of each MT cell appears to be influenced by a handful of glomeruli that are spatially sparse and can be very distant^25^. Diversity across M72-MT cell responses to a single odor could therefore result from each cell having different connectivity within the lateral network.

**Fig. 7:**
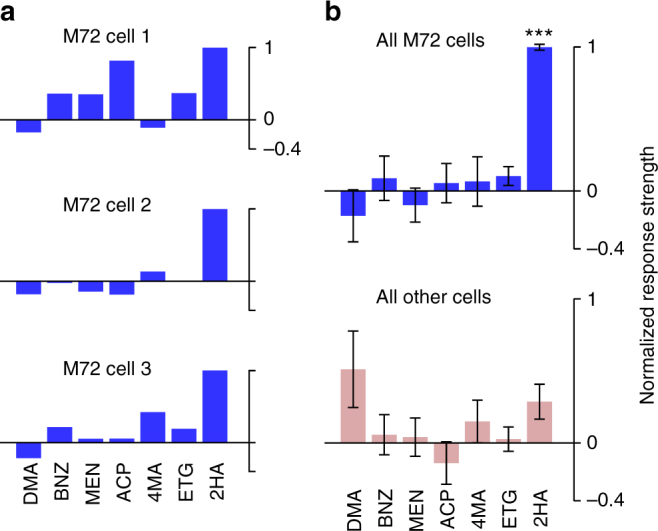
Odor-evoked rate responses display stereotypy also only for the strongest ligand. **a** Odor response profiles of three M72-MT cells, calculated as the mean change in spike count over the duration of the entire first sniff. Each cell’s responses are normalized such that 0 (horizontal line) is the spike count during baseline (air) and 1 is the maximum spike count across odors. **b** Odor response profiles averaged across the population; for M72-MT cells (top) and generic MT cells (bottom). M72-MT response is significantly higher than the generic MT response only for 2HA (Wilcoxon signed-rank test). Error bars = SEM

Is this diversity/stereotypy phenomenon unique to the M72 glomerulus, or is it a general feature of glomerular channels of the olfactory bulb? Previous observations in an anesthetized preparation suggest that the effect we observe exists for another glomerulus. Tan et al.^8^ recorded firing rate responses of MT cells receiving input from the I7 odorant receptor. They showed that the strongest known ligand for that receptor consistently evoked high spike counts in I7 MT cells, but other odorants (not necessarily ligands for I7) typically did not. When we analyzed our data using their method, we observed the same pattern (Fig. 7), implying that this phenomenon may be a common feature of all glomerular channels in the active, functioning bulb. Moreover, we revealed a temporal specificity of this effect, namely that the consistent response to a strong ligand manifests as a temporally stereotyped, early excitatory transient.

Dhawale et al.^10^, working in anesthetized mice, found that sister MT cells typically responded to an odor with substantial temporal diversity, yet responded coherently to the common drive provided by optogenetic stimulation of the parent glomerulus. By studying a glomerulus with functionally defined inputs, we show for the first time that response diversity among sister MT cells exists even for known ligands of the parent glomerulus, and that a coherent response can in fact be elicited by an odor stimulus. We posit a parallel between activation of a glomerulus with a strong ligand and artificial optogenetic stimulation—in both cases, the specific glomerular excitation could be transmitted to MT cells unperturbed by preceding activity in other channels, thereby producing synchronous activity in the corresponding sister MT cells.

**Fig. 8:**
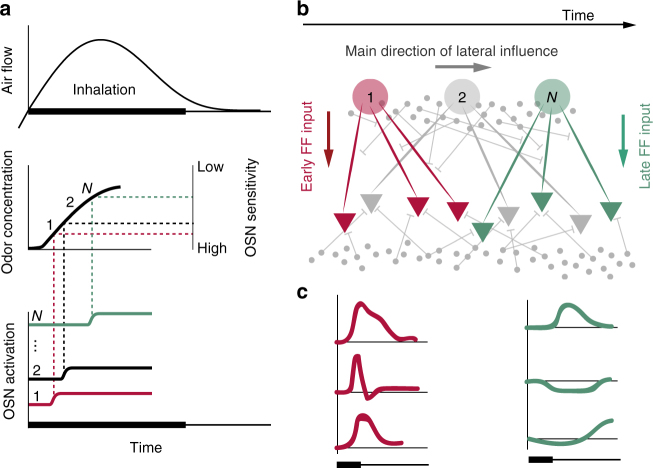
A proposed mechanism responsible for stereotypy/diversity among sister MT cell odor responses. **a** In the presence of a given odor, OSN receptors’ relative sensitivities to the odorant determine the relative response latencies of their corresponding glomeruli. As inhalation carries the odor into the nose (top), odor concentration rises gradually (middle, left). More sensitive olfactory receptors reach their activation threshold earlier (middle, right), and thus respond earlier (bottom). The labels 1 to *N* denote the temporal order of OSN channel activation. **b** The ensuing flow of activity in the bulb. The schematized olfactory bulb network is shown as in Fig. 1: glomeruli (large colored circles); MT cells (colored triangles); and inhibitory neurons (small gray circles; top = periglomerular layer, bottom = granule layer). Thin gray lines represent inhibitory connections. The glomeruli are depicted horizontally in the order of the temporal sequence of activation (1 to *N*), as shown in **a**. MT cells connected to glomerulus 1 (red) receive early feedforward (FF) excitation, which propagates through the inhibitory network. MT cells connected to glomerulus *N* (green) receive both FF excitation and lateral inhibition. **c** Responses of MT cells connected to early-activated (red) and late-activated (green) glomeruli. Driven by a common excitatory input, MT cells connected to early-activated glomeruli share an initial short-latency excitatory response. MT cells connected to later-activated glomeruli are subject to both excitatory drive and heterogeneous inhibitory influences, and thus show diverse responses

Assuming universality of the observed phenomenon, what could underlie the observed patterns of diversity and stereotypy? We propose that the relative timing of activity entering the olfactory bulb could be responsible for the observed patterns of diversity and stereotypy in sister MT cells. When an odorant is presented, glomeruli corresponding to the highest-affinity receptors may be excited first, while those with lower affinities may be activated later (Fig. 8a). Such an activation sequence could result from several mechanisms: (1) a gradual rise in odorant concentration during inhalation, over tens to hundreds of milliseconds, resulting in different OSN types reaching threshold at different times^26,27^; and (2) a cellular signal integration process by which OSNs activated by stronger ligands reach firing threshold faster^28^. The earliest activated glomeruli would confer excitatory drive to downstream sister MT cells, which would further propagate signals into downstream inhibitory networks. This inhibitory activity will then feed back to the population of all MT cells in different ways (due to the heterogeneity of lateral connectivity), thus diversifying responses in subsequently activated glomerular channels (Fig. 8b). For a given odorant, the net effect of these feedforward and recurrent dynamics will thus be different for MT cells in early- and late-responding channels. The initial input experienced by MT cells of early-responding channels will be dominated by feedforward excitation, causing these cells to produce a stereotypical burst of action potentials early in the sniff cycle (Fig. 8c). Sister MT cells associated with late-responding channels will receive heterogeneous inputs from the inhibitory network coincidentally with the feedforward excitation, resulting in diverse responses.

The results of our experiment are consistent with this hypothesis. Although we do not know the exact timing of M72-OSN activation relative to other channels for each odor, the fact that 2HA is such a strong ligand for the receptor suggests that the M72 channel is one of the first to be activated in response to this odor. Conversely, the relatively weak sensitivity of M72 receptors to the other ligands predicts that the M72-OSNs would be activated relatively late.

Moreover, this model provides a simple explanation for why these results do not change with odor concentration. As odor concentration decreases, OSNs are typically activated later^4^. However, decreasing odor concentration would not change the relative timing of glomerular activation within our model. The MT cells connected to later-activated glomeruli would receive diverse and concentration-dependent inhibitory drive; thus, sister MT cells would still respond to the odor differently from one another, but also would show variable responses across concentration (Figs. 5 and 6).

Our data are consistent with this hypothesis, but there are a few limitations to our conclusions. First, showing that the relationship between diversity/stereotypy and odor affinity holds for all glomeruli would require recording from MT cells across many different channels that have known high-affinity ligands, and testing odorants across concentration—currently such experiments are technically very challenging.

Second, gauging the relative sensitivity of all odorant receptors to a given odorant, or the temporal sequence of glomerular activation in vivo, is also quite challenging. Here we used the inverse approach, assuming that the relative sensitivity of M72 receptors to multiple odorants is a proxy for the temporal ordering. It is possible that other receptors could have an even higher affinity to 2HA than M72. It is tempting to assume that MT cells should respond earlier for a higher-affinity ligand than for an intermediate or weak ligand. While this relationship is observed in our data (Supplementary Fig. 6), the absolute latency of response to a given ligand likely depends not only on receptor affinity but also on the physical and chemical properties of the ligand. For example, a high concentration of a hydrophilic ligand may evoke earlier responses than a weak concentration of a hydrophobic ligand. Thus, we avoid drawing conclusions about latency differences across different odors, and focus instead on latency differences for a given odor between cells.

Third, our methods may introduce sampling biases. It is possible that we are only recording from a subset of M72-MT neurons. Our optogenetic technique only identifies MT cells that receive a dominant feedforward excitatory connection from the M72 glomerulus; this, however, accounts for a particularly relevant subset of cells in conveying odor information. It is also plausible that a small fraction of our putative M72-MT population is made up of granule cells, although we consider this unlikely due to their location and size relative to MT cells^29^.

And lastly, while validation of the mechanism proposed above is beyond the scope of this report, our model provides a set of hypotheses. For instance, under this model, blocking inhibition (optogenetically or pharmacologically) would increase the number of odors evoking a stereotyped response in sister MT cells. Future experiments are needed to explore this possibility and the mechanisms underlying the conditions of diversity and stereotypy in MT output.

How might stimulus-dependent stereotypy be incorporated into a broader olfactory code? We imagine a downstream decoder that is particularly sensitive to synchrony between sister MT cells of a common glomerulus. Such a decoder need not depend on a topographical map between the olfactory bulb and the piriform cortex as it could be implemented through random projections^30^. For strong ligands, sister MT cells will all respond with a relatively short-latency excitatory transient, and the decoding neuron will thus fire as well. For weaker ligands, the temporal diversity amongst sister MT cells would fail to provide sufficient coherent input to drive the decoding neuron. In such a scheme, lateral inhibition preserves information in early-responding channels and scrambles information in late-responding channels. This configuration would act to sharpen and sparsify the odor representation, reducing the dimensionality of the peripheral combinatorial code to one that is dominated by the most sensitive glomerular channels. Moreover, our observation that the temporal stereotypy/diversity of responses is robust to changes in concentration means that the readout representation would also be concentration-tolerant, and thus could encode odor identity independent of concentration. This model is consistent with a recently proposed primacy coding hypothesis^31^, which emphasizes the role of the most sensitive glomeruli as those responsible for concertation invariant odor identification. The stereotypy of MT cell responses driven by strong ligands is also consistent with recent observation of concertation invariant early odor responses of cortical cells^32^. The phenomenon of diversity/stereotypy of MT cell responses described here may provide a mechanism by which olfactory bulb circuitry could implement a primacy coding model.

## Methods

### Animals

For electrophysiological experiments, we used adult homozygous *M72-IRES-ChR2-YFP* mice (strain *Olfr160*^tm1.1(COP4*/EYFP)Tboz^). Data for MT cell in vivo odor responses were collected in 17 animals (13 males and 4 females). Animals were 6–10 weeks old at the beginning of the experiment and were maintained on a 12-h light/dark cycle (lights on at 20:00 h) in isolated cages in a temperature- and humidity-controlled animal facility. All animal care and experimental procedures were in strict accordance with protocols approved by the New York University Langone Medical Center and Northwestern University Institutional Animal Care and Use Committees.

### OSN electrophysiology

Perforated patch recordings were made from the dendritic knobs of fluorescently labeled M72-expressing OSNs as described previously^11,13,33^. In short, the olfactory epithelium from neonatal mice was removed and kept in oxygenated artificial cerebrospinal fluid (95% O_2_ and 5% CO_2_), containing 124 mM NaCl, 3 mM KCl, 1.3 mM MgSO_4_, 2 mM CaCl_2_, 26 mM NaHCO_3_, 1.25 mM NaHPO_4_, and 15 mM glucose, pH 7.4, 305 mOsm. The epithelium was transferred to a recording chamber at 20–23 °C and imaged using an upright fluorescence IR-DIC microscope equipped with a charge-coupled devise (CCD) camera and a 40× water-immersion objective. Perforated patch clamp was performed by including 260 μM amphotericin B in the recording pipette, which was filled with 70 mM KCl, 53 mM KOH, 30 mM methanesulfonic acid, 5 mM EGTA, 10 mM HEPES, and 70 mM sucrose, pH 7.2, 310 mOsm. The electrodes had tip resistances ranging from 8 to 10 MΩ, and liquid junction potentials were corrected in all experiments. Signals were acquired at 10 kHz and low-pass filtered at 2.9 kHz. Odorants were applied via pressure ejection via a multi-barrel pipette placed 20 μm downstream of the cell. Odorants were dissolved in dimethyl sulfoxide and diluted in bath solution to achieve desired concentrations.

### Gene targeting

OMP-GCaMP3: The coding sequence of GCaMP3^33,34^ was flanked by AscI sites and cloned into a targeting vector for the olfactory marker protein (*omp*) locus^34,35^ so that the coding sequence of OMP is replaced by that of GCaMP3, followed by a self-excising neomycin selection cassette^35,36^. The targeting vector was electroporated into a 129 ES line, and clones were screened for recombination by long-range PCR. Chimeras were generated from recombinant clones by aggregation with C57BL/6 embryos.

M72-RFP (M72-IRES-tauCherry): A cassette containing an internal ribosome entry site (IRES), followed by the coding sequence for a fusion of bovine tau and mCherry^12,36^ and a self-excising neomycin selection cassette, was inserted into an AscI site located three nucleotides downstream of the M72 coding sequence in an M72 (*olfr160*)-targeting vector^12^. The targeting vector was electroporated into a 129 ES line, and clones were screened for recombination by long-range PCR. Chimeras were generated from recombinant clones by injection into C57BL/6 blastocysts.

### Olfactory bulb imaging

Awake in vivo imaging: Imaging was done in 7- to 8-week-old naive male mice that were heterozygous for the OMP-GCaMP3 and homozygous for the M72-RFP allele, and that had been implanted with chronic optical imaging windows and head bars. Mice were first anesthetized with isoflurane (2–3%) in oxygen and administered buprenorphine (0.1 mg kg^−1^) as analgesic; bupivacaine (2 mg kg^−1^) as a local anesthetic at the incision site; and dexamethasone (2 mg kg^−1^) to reduce cerebral edema. The animal was secured in a stereotaxic head holder (Kopf instruments) and the bone overlying the olfactory bulbs was thinned to transparency using a dental drill. Two micro-screws were placed into the skull to structurally support the head bar. A custom-built titanium head bar (3 mm × 15 mm, <1 g) was attached to the skull using Vetbond cyanoacrylate glue and cemented in place using dental cement (Dental Cement, Pearson Dental Supply). Black Ortho Jet dental acrylic (Lang Dental Manufacturing) was extended from the head cap around the thinned bone forming a small chamber. The area overlying the olfactory bulbs was covered with multiple thin layers of prism clear cyanoacrylate glue (Loctite #411) as described^11,13,34^.

Following complete recovery from surgery, mice were placed on a water restriction schedule (1 ml per day). After 7–10 days of water restriction, mice were slowly habituated to the imaging setup where they were trained to lick for a water reward. During imaging sessions, mice were positioned on a custom-built wheel and secured with the head bar in a custom-built holder.

Light excitation was provided using a 200 W metal-halide lamp (Prior Scientific) filtered through standard filters sets for RFP (49008, Chroma) and green fluorescent protein (GFP; 96343, Nikon). Optical signals for GCaMP were recorded using a CCD camera (NeuroCCD SM256; RedShirtImaging) at 25 Hz with a ×4 temporal binning. Each recording trial was 16 s consisting of a 6 s pre-stimulus interval, a 4 s odor pulse, and a 6 s poststimulus interval. Only one odorant per day was tested to avoid cross contamination of different odorants. Different concentrations of the same odorant were interleaved with clean air trials to identify potential contamination.

### Response maps

Response maps were obtained by temporally averaging the response signal over a 0.5 s window around the time of maximum response, and subtracting a pre-stimulus response baseline (1.6 s window). For low concentrations, stimuli were presented at least three times and averaged to obtain response maps. Images were processed and analyzed in Neuroplex (RedShirtImaging) and Image J (NIH) software.

Response amplitudes were measured from a region of interest drawn around the M72 glomerulus in the RFP image. Only the first trial of each odor concentration was used to obtain the response amplitude to avoid potential adaptation effects.

### Implantation surgery

Mice were anesthetized using isofluorane gas anesthesia. A diamond-shaped bar for head fixation^37^, a reference electrode, and a pressure cannula for sniff recording^6^ were implanted. To implant the sniffing cannula, which was a thin 8.5-mm-long stainless capillary (gauge 23, Small Parts capillary tubing), a small hole was drilled in the nasal bone, into which the cannula was inserted, fixed with glue, and stabilized with dental cement. The reference electrode was implanted in the cerebellum. The mice were given at minimum 5 days after surgery for recovery.

### Setup and odor habituation

After recovery, mice entered a regime of water restriction, with 1 ml administered every day. Five days into this regime, the mice were placed in a head-fixation setup for lick training^6,17,37^. To reduce stress to the animals and movement artifact during recordings, mice were positioned on a running wheel. Mice could stand still or walk on the wheel as desired. The first few sessions were brief (10–20 min) and served purely to acclimate the animals to head fixation and the running wheel. Mice typically remained mostly quiescent after one to two sessions of head fixation, after which lick training sessions began. A lick spout was placed in front of the animal, which delivered a droplet of water every time the animal licked it. Mice typically learned the water-rewarding nature of the head-fix setup within one to three sessions. We then removed control of the water delivery from the mouse and started delivering one out of seven odors in pseudo-random sequence, with an average inter-stimulus interval of 8 s and stimulus duration of 1000–4000 ms. A drop of water was delivered to the mouse automatically every three to five odor presentations. Animals underwent three to five sessions of odor exposure (200–400 trials each) of this type before recordings. This procedure served several purposes: (i) it reduced the distress of mice in the setup; (ii) it reduced the movement artifact during recordings; and (iii) it habituated the animals to the set of odorants used in the experiment, thus eliminating any novelty effects.

### Water delivery

Water delivery was based on gravitational flow controlled by a pinch valve (98302–12, Cole-Parmer) connected via Tygon tubing to a stainless steel cannula (gauge 21, Small Parts capillary tubing), which served as a lick tube. The lick tube was mounted on a micromanipulator and positioned near the mouse’s mouth. The water volume was calibrated to give approximately 2.5 μl per valve opening. Licks were detected by the closing of an electrical circuit through the grounded mouse (the circuit was open until the mouse connected the metal cannula to ground).

### Behavioral and stimulus delivery control

All behavioral events (odor and final valve opening, laser stimulation, water delivery, and lick detection) were monitored and controlled by a real-time (1 ms), Arduino platform-based, behavioral controller box, developed at Janelia Farm Research Campus, HHMI. In each trial, the behavioral controller read trial parameters, and sent trial results together with a continuous sniffing signal to a PC running a custom-written Python program, Voyeur (partially developed by Physion Consulting, Cambridge, MA). Voyeur is a trial-based, behavioral experiment control and acquisition software that allows behavioral protocols to compute parameters of trials and send them to embedded real-time hardware systems. The Arduino code and Python application source is available as a GitHub repository (search for Voyeur in GitHub). Every stimulus and behavioral event had an associated trigger signal that was sent to the recording system for precise synchronization with neural activity recordings.

### Sniff recording

To monitor the sniff signal, the implanted sniffing cannula was connected to a pressure sensor through an 8–12 cm-long polyethylene tube (801000, A-M Systems). The pressure was transduced with a pressure sensor (24PCEFJ6G, Honeywell) and homemade preamplifier circuit. The signal from the preamplifier was recorded together with electrophysiological data on one of the data acquisition channels. The timing of the pressure signal was calibrated with a hot wire anemometer (mini CTA 5439, Dantec Dynamics, Denmark) as in Shusterman et al.^6^. The time differences between pressure signal and the flow signal during calibration did not exceed 2–3 ms. The cannula was capped when not in use.

### Light stimulation

Light stimulation was produced via a 100 µm multimodal fiber coupled to a 473-nm diode laser (model FTEC2471-M75YY0, Blue Sky Research). The end of the fiber was cut flat and polished. The light stimulus power at the open end was measured by a power meter (Model, PM100D, Thorlabs), and calibrated to adjust the amplitude of the voltage pulses sent to the laser, to achieve a consistent power output across experiments.

### Odor delivery

For odor stimulus delivery for electrophysiological experiments, we used an eight-odor air dilution olfactometer. Approximately 1 s prior to odor delivery, a stream of nitrogen was diverted through one of the odorant vials at a rate between 100 and 10 ml min^−1^, and then merged into a clean air stream, flowing at a rate between 900 and 990 ml min^−1^, thus providing 10- to 100-fold air dilution. Gas flows were controlled by mass flow controllers (Alicat MC series) with 0.5% accuracy. The odorized stream of 1000 ml min^−1^ was homogenized in a long thin capillary before reaching the final valve. Between stimuli, a steady stream of clean air with the same rate flowed to the odor port continuously, and the flow from the olfactometer was directed to an exhaust. During stimulus delivery, a final valve (four-way Teflon valve, NResearch, SH360T042) switched the odor flow to the odor port, and diverted the clean airflow to the exhaust (Supplementary Fig. 7). Temporal odor concentration profile was checked by mini photoionization detector (PID) (Aurora Scientific, model 200B). The concentration reached a steady state 95–210 ms (depending on a specific odor) after final valve opening. To minimize pressure shocks and provide temporally precise, reproducible, and fast odor delivery, we matched the flow impedances of the odor port and exhaust lines, and the flow rates from the olfactometer and clean air lines. As sniff activity was monitored in real time, the final valve was activated at the onset of exhalation, so that the odor reached steady-state concentration before the next inhalation. At the end of the odor delivery (duration 1–4 s) the final valve was deactivated, and the nitrogen flow was diverted from the odor vial to an empty line. Inter-odor delivery interval was 7–14 s, during which clean air was flowing through all Teflon tubing.

All odorants (see Supplementary Table 1, purchased from Sigma-Aldrich) were diluted in mineral oil and stored in liquid phase in dark vials. The level of dilution of each odorant was estimated to achieve equal concentrations for all odorants of 0.075 + 0.01 µM after 10-fold air dilution^11,13,38^. Each vial contained 5 ml of mineral oil with diluted odorant and 45 ml of headspace.

For concentration series experiments for two odorants, 2HA and MEN, we changed the dilution level across approximately two orders of magnitude. The final desired concentrations were calibrated daily, immediately before the experiment began, and were achieved by tuning the air dilution and matching PID signals between vials with different liquid dilutions.

The odor delivery system for imaging experiments was almost identical. However, due to differences in dilution procedure the matching concentration for 2HA was 2× higher in the imaging setup than in electrophysiological experiments, and for MEN the matching concentration was 1.8× lower.

### Olfactory bulb electrophysiology

MT cell spiking activity was recorded using 16- or 32-channel Si-probes (NeuroNexus, model: a2x2-tet-3mm-150-150-121(F16), Buzsaki32(F32)). Cells were recorded in the dorsal mitral cell layer. The identity of MT cells was established on the basis of criteria formulated in previous work^10,39^ (while we cannot rule out granule cells, it is unlikely that we recorded from them with our extracellular technique, based on their location and significantly smaller soma size^29^). The data were acquired using a 32-channel data acquisition system (HHMI Janelia Farm Research Campus, Applied Physics and Instruments Group,) with widely open broadband filters and sampling frequency of 19 531 Hz.

### Recordings

Initial preparation: At the beginning of a recording session, a mouse was anesthetized with gas isofluorane and placed in the head-restraint setup. The running wheel was locked and a heating pad was placed under the animal. The lateral M72 glomerulus in either the right or left olfactory bulb was located using a fluorescent dissecting microscope, and the overlying bone was thinned. The open end of the fiber used for optical stimulation was positioned above the glomerulus, making contact with the thinned bone but without pressing on it.

A craniotomy was made just medial to the glomerulus, the dura removed, and the silicon probe was inserted at an angle (25–45° from vertical), driven by a digital micromanipulator (MP-285, Sutter Instruments). The insertion point was chosen so that at a depth of ~300–500 µm from the brain surface, the tip of the most posterior shank in the probe would be roughly in line with the glomerulus in the medial/lateral axis. The anterior/posterior position was varied (following anatomical data from Liu and Urban, unpublished).

Search for MT cells putatively connected to M72 glomerulus: The anesthesia was removed and once the animal awakened, the probe was lowered to the external plexiform layer and advanced at ~5 µm intervals. At each position, a light pulse (0.5–15 mW power, 1–2 ms duration) was delivered to the glomerulus, triggered on the onset of inhalation. The peri-stimulus activity on all sites of the Si-probe was monitored. A spiking increase with short latency after the light pulse (below 20 ms, typically 5–10 ms) indicated the presence of a cell receiving input from the stimulated glomerulus. If no light-responsive cells were found upon reaching a depth of ~700–800 µm, the electrode was raised, reinserted, and the search repeated.

Recording odor responses: After locating a putative M72-MT cell, odor recording session was initiated. Multiple odorant stimuli with fixed concentrations or two odorant stimuli with multiple concentrations were presented pseudo-randomly with 7–14 s inter-trial interval. After every 2–4 odor trials, a light pulse was delivered to re-confirm the presence of the M72-MT cell. For each odor stimulus, 20–35 trials were collected.

All sites of the Si-probe were used to monitor activity of other, non-light-responsive units, during M72-MT recording sessions. In addition, to increase the pool of non-M72-MT cell (other cells), we anesthetized the animal again, performed a new craniotomy, and placed the probe at a new site, usually further anterior, and performed recordings with the same stimulus set.

### Spike extraction

Acquired electrophysiological data were filtered and spike sorted. We used the Klusta suite software package for spike detection and spike sorting^6,40^ and software written by E.M.A. and D.R.

### Identification of M72-MT cells

We defined MT cells functionally connected to the M72 glomerulus as units that displayed an excitatory, short-latency response to light stimulation (1 ms, 5–15 mW) of the ChR2-expressing M72 glomerulus. While in general it is difficult to establish monosynaptic connectivity using optogenetic stimulation^41^, we capitalized on the known anatomy of the olfactory bulb: MT cells receive excitatory input from a single glomerulus, and interactions between MT cells connected to different glomeruli are inhibitory^3,42^.

We compared the PSTHs of MT cells with and without light stimulation. PSTHs with 4 ms temporal bins were referenced to the onset of inhalation at the onset of inhalation, when the light stimulation was presented. The MT cell was considered light responsive if light-evoked activity exceeded activity in the no-light condition by at least one standard deviation, in at least one 4 ms temporal bin, within 50 ms after the onset of the light pulse. The latency was estimated as the first time point when such a deviation occurred. The distribution of latencies is shown in Fig. 1e. The majority of the responses (1.5 interquartile range (IQR)) occurred with latencies shorter than 22 ms. Two cells responded with latencies larger than 1.5 IQR, 22 ms, and were removed from the pool of cells used in this study.

### Recording of generic MT cells

Most generic cells were recorded simultaneously with the M72-MT cells, and identified as the ones that did not respond to light stimulation of the M72 glomerulus. Note that the distance between the shanks ranges from 150 to 600 μm, and that the range of inhibitory connections within the MT cell networks has been found to be spatially sparse and long and heterogeneous in range. Occasionally, penetrations were done at a random location.

### Estimation of unit depth

We identified the group of sites of the array in which the unit was detected, and estimated its centroid. We then computed its distance to the tip of the probe, corrected by the angle of insertion to project to the dorsal/ventral axis. The position of the tip of the probe (dorsal/ventral) was kept track of and recorded during the experiment, relative to the surface of the brain at insertion point.

### Estimation of mean firing rate and preferred phase of spontaneous spiking

We computed the mean firing rate of the cell across sniff cycles prior to odor presentation. We estimated the preferred phase of the spontaneous firing as the time when the baseline (no odor) snifflet rate was maximum.

### Snifflet analysis of the response profiles

We built probabilistic models to describe the encoding of odor stimuli by MT neurons. These models take the form of Generalized Linear Models^43,44^, and describe a generative model for the spiking data. For a given cell and a given odor (or the baseline condition, with no odor presentation), we assumed that the firing rate of the cell changes over the course of the sniff according to a specific temporal pattern, which we call a “snifflet”. Given that the observed spiking patterns during individual sniffs depend on the inhalation duration (Fig. 2d), we built this dependency into the model. In particular, we assumed that during an individual sniff, the firing rate is generated by temporally dilating the snifflet by a sniff-dependent factor. The value of this dilation factor, *α*, depends on the duration of the inhalation phase of that sniff. More formally, we write the rate *r*(*t*) as

$$r\left( t \right) = {\mathrm{exp}}\left[ {\mathop {\sum }\limits_{i = 1}^n \psi \left( {\alpha _{i}\left( {t - \tau _{i}} \right)} \right) \cdot {\mathrm{{\Pi}}}\left( {\tau _{i} \mathrm{\le} t {} \tau _{i + 1}} \right)} \right]$$

where *ψ*(*t*) is the odor-evoked snifflet, *α*_*i*_ is the temporal dilation factor for the *i*^th^ sniff, *τ*_*i*_ is the onset time of the *i*^th^ sniff, *n* is the total number of sniffs and ${\mathrm{{\Pi}}}\left( \cdot \right)$ is an indicator function, such that the response pattern is reset at the onset of the next sniff. Removing the indicator function, so that snifflets from successive odors overlap, does not qualitatively change any of the major results in the main text.

The model requires a choice of dilation factors, *α*_*i*_, for each sniff. Motivated by the work of Shusterman et al.^6^, we fixed the dilation factors as the reciprocal of the inhalation durations, $\alpha _i = 1/d_i^{\mathrm{inh}}$, where $d_i^{\mathrm{inh}}$ is a duration of the inhalation phase of the sniff cycle. This produced better model fits than alternative choices, such as dilating with the reciprocal of the full sniff durations, separate dilation for the inhalation and rest-of-sniff components of the response, or fixing *α*_*i*_ = 1 (i.e., no temporal dilation) throughout (Supplementary Fig. 4).

The free parameters of the model are the snifflet time course, *ψ*(*t*), for each cell and odor. We parameterized the snifflets as a length-*KD* vector (for integers *K* and *D*), such that the first *D* components represent the evolution of the cell’s firing rate during the inhalation period, and the remaining (*K* − 1)*D* components represent the evolution of the cell’s firing rate during the remainder of the sniff. The integer *D* thus defines the sampling resolution for the snifflet, and *K* the relative duration of post-inhalation response to model. We use *D* = 30 and *K* = 4 in the main text, but other values produced similar results.

We also placed priors over the components of *ψ*(*t*), to constrain the snifflets would evolve smoothly in time. We used the Automatic Smoothness Determination prior^45^ and learned the hyper-parameters via evidence optimization^16,46^. Including the prior dramatically increased the quality of model predictions on held-out data (Supplementary Fig. 4).

We solve for *ψ*(*t*), by maximizing its posterior probability. Given a point estimate of the hyper-parameters, and using a fixed scheme for determining $\alpha$ (above), this is a convex problem^15^, which we solve using conventional Newton methods. We approximate the posterior on *ψ*(*t*) using a Laplace approximation. For the purposes of illustration, we show the snifflets in the main text in their exponentiated form (i.e., in terms of firing rate, rather than log firing rate). Where error bars on individual snifflets are shown (Fig. 2e), the shaded areas illustrate only the marginal variance of the approximate posterior at each time point, rather than the joint covariance across time. Statistical comparisons between odor-evoked and baseline snifflets (Figs. 4 and 5) were performed in log firing rate space; we again consider only the marginal variance at each time point.

The snifflet model provides a parameterization of how inhalation duration (a nuisance variable) affects spiking response, allowing us to factor this relationship out from our results and study the differences across odors and cells. To verify that our results did not depend on the particulars of the snifflet model, we fitted the spiking data without adjusting for variations in sniff duration (Supplementary Fig. 5).

### Mouse strain availability

OMP-GCaMP3 and M72-RFP strains will be made available through The Jackson Laboratory (Stock #029581 and #029637).

### Code availability

Code developed for this work is available in the following github repositories: https://github.com/admiracle/Voyeur (stimulus delivery system control); https://github.com/zekearneodo/ephys-tools (post-recording data preparation, pre-processing and initial sniff analysis); and https://github.com/rabbitmcrabbit/snifflet (snifflet analysis).

### Data availability

The electrophysiological dataset is available at 10.6084/m9.figshare.5877474. The complete datasets generated and/or analyzed during the current study are available from the corresponding author on reasonable request.

## Electronic supplementary material

Supplementary Information
