# Supplementary material for: Stimulus dependent diversity and stereotypy in the output of an olfactory functional unit
Source: Nat Commun. 2018 Apr 9;9:1347. doi: 10.1038/s41467-018-03837-1 (PMC5890244; doi:10.1038/s41467-018-03837-1)
Supplement: Supplementary file 1 — Supplementary Information [file 41467_2018_3837_MOESM1_ESM.pdf]

Supplementary Table 1

Odorant	Notation	*EC_{50}* (μM)
2-hydroxy acetophenone	2HA	0.03 ± 0.01
ethyl tiglate	ETG	1.4 ± 0.2
4-methyl acetophenone	4MA	1.4 ± 0.3
acetophenone	ACP	13.9 ± 3.8
menthone	MEN	17.3 ± 5.1
benzaldehyde	BNZ	18.6 ± 1.8
2,4-dimethyl acetophenone	DMA	36.3 ± 3.2

List of odorants used in all experiments, with their abbreviations and EC_{50} values (obtained from M72-OSN patch recordings; see main text).

Supplementary Figure 1

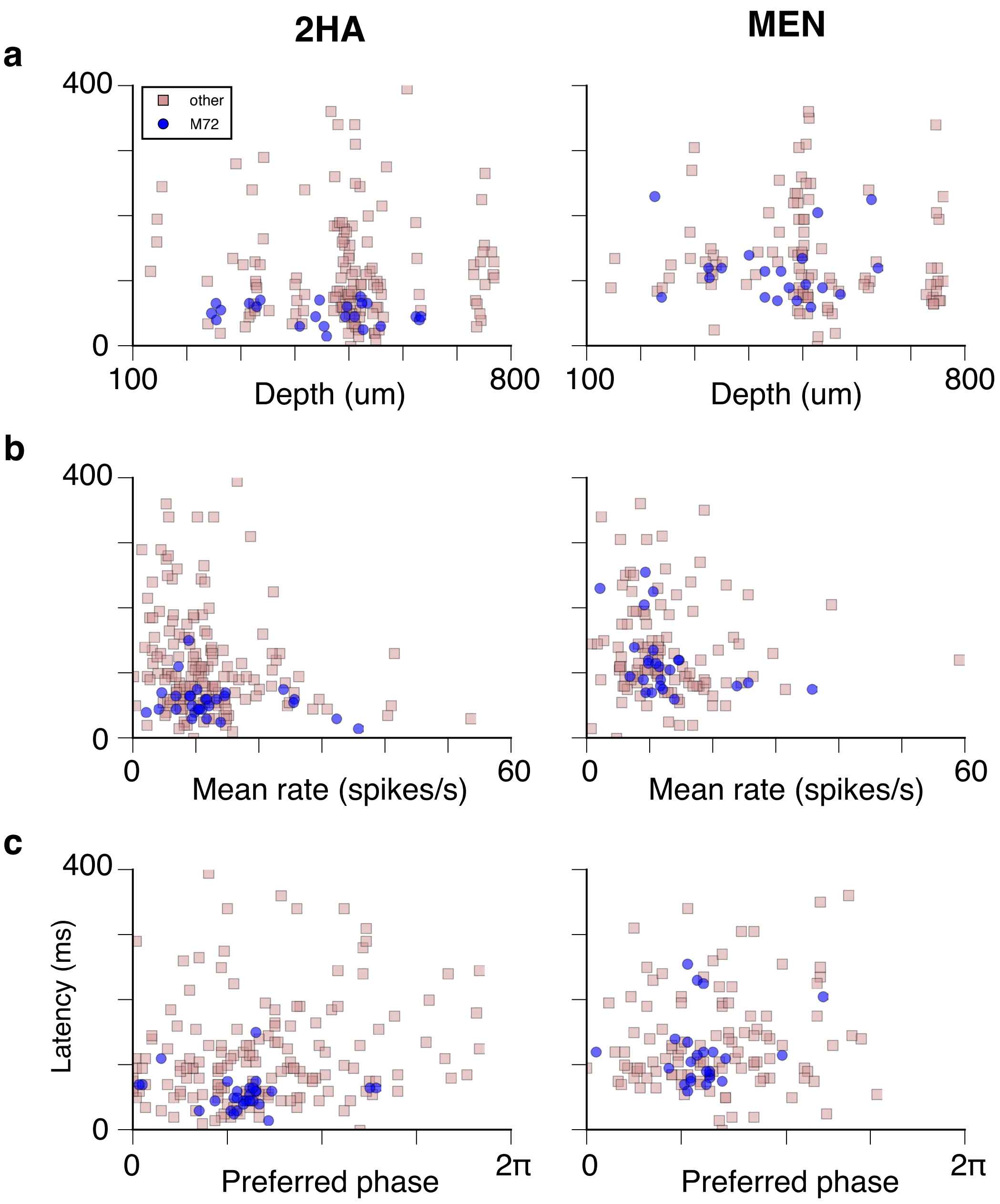

The response latencies of M72-MT cells (blue) and generic MT cells (pink) are plotted against approximate cell recording depth (a), mean spontaneous firing rate (b) and preferred sniff phase of firing (c), for two odors 2HA (left) and MEN (right). See Methods section for details.

Supplementary Figure 2

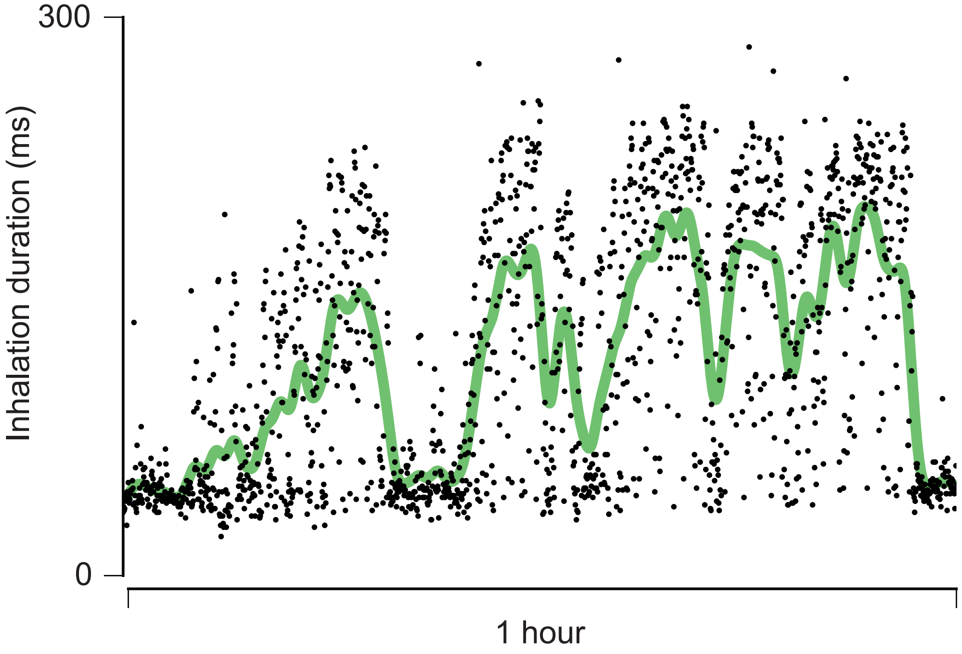

Example of the pattern of sniffing of an individual animal over an hour. Black dots show the inhalation duration of successive sniffs during one recording session; thick green line shows a temporally smoothed average of inhalation duration. The rarer fast sniffs predominantly occur during isolated periods, which here can be seen at the beginning and end of the session, and at about 20 minutes in.

Supplementary Figure 3

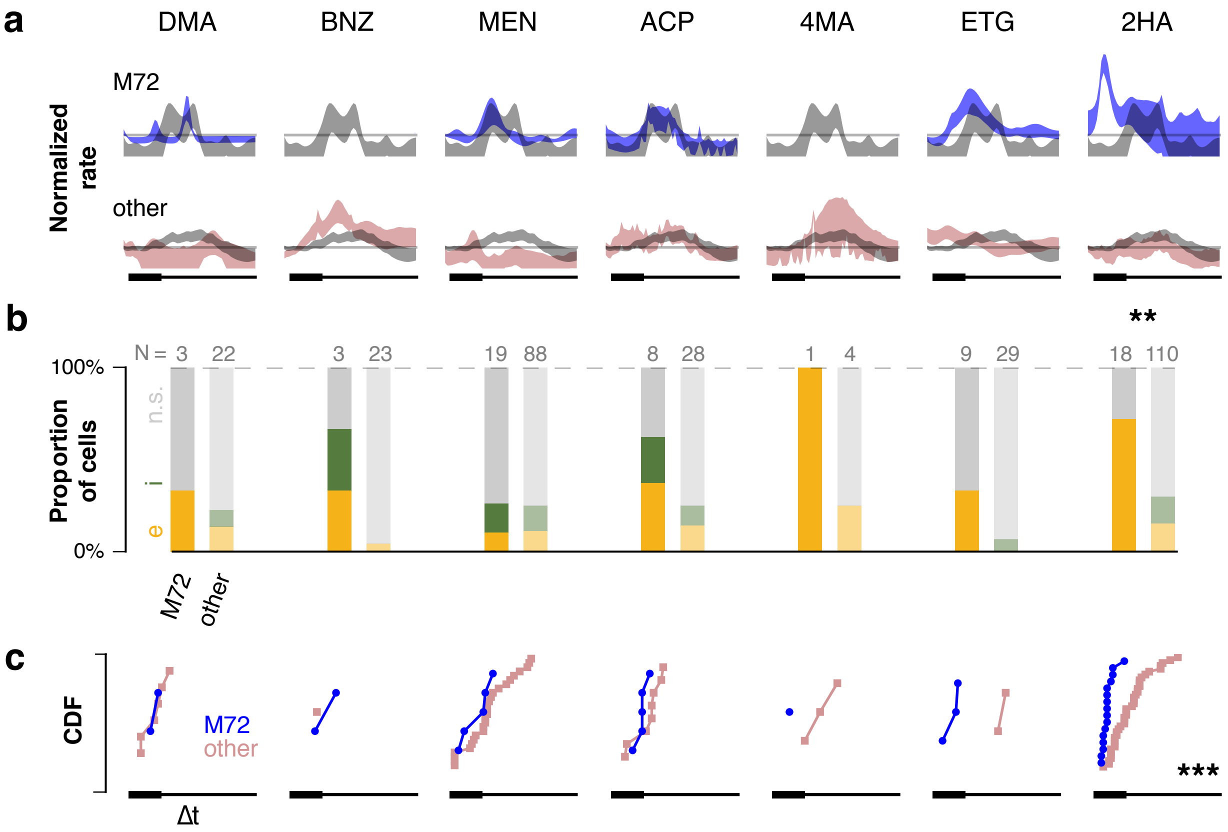

Same analyses and results as shown in Fig. 4, here including only fast sniffs (inhalation durations shorter than 100 ms). **(a)** Mean snifflet across M72 (blue) and other (pink) MT cells, for each odor. **(b)** Distributions of the polarity of the first significant response of cells to each odor (e: excitatory; i: inhibitory; n.s.: not significant), compared between the M72 (bolded bars) and other (desaturated bars) MT sub-populations recorded. **(c)** Cumulative distributions of the latency of the first significant response, for M72 (blue) and other (pink) MT cells. Fast sniffs were rarer (<25%), and seemed to occur as part of a distinct behavioral state of the animals (Supplementary Fig. 2). We therefore focused exclusively on slower sniffs in the main text. Here we repeat the analysis of Figure 4 on the fast sniffs only. As fast sniffs were rare for many animals, there was not enough data to analyze for most cells. However, the same trends can be observed as in Fig. 4. The number of fast sniffs during the concentration experiment was even smaller, preventing a comparable analysis for Fig. 5 during fast sniffs.

Supplementary Figure 4

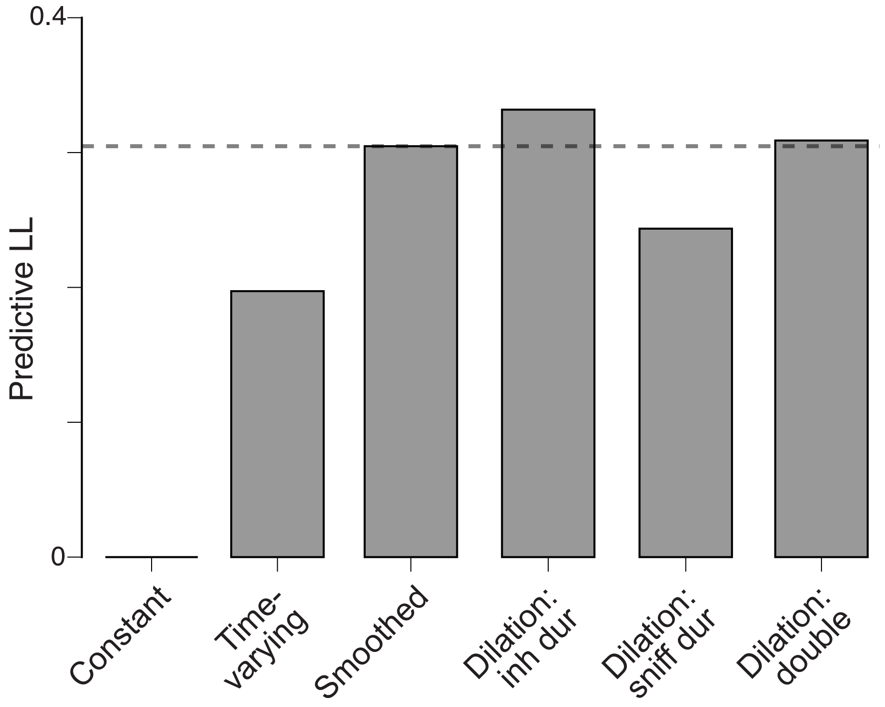

Quality of model fits, measured as log likelihood (LL) on held-out data. In the main text, we describe the fitting of snifflet models to odor-evoked responses of MT cells. Here we quantify the quality of these fits, and justify the modeling decisions made. Bars show the predictive LL (per second) on held-out data of the fitted models, averaged across MT cells and odors. Note that these scores are cross-validated: improvements indicate that the models are explaining structure in the data, rather than overfitting. Values are expressed relative to the constant model (left), i.e. where the firing rate of each neuron is assumed to be constant over the duration of the sniff. The other bars show four snifflet models. *Time-varying*: odor responses described via an unsmoothed PSTH with a ridge prior, and no temporal dilation. This shows the performance of a naive, sniff-aligned model of odor-evoked responses. *Smoothed*: Same, but with a smoothed PSTH, with a learned smoothing time constant for each cell/odor. *Dilation: inh dur*: the smoothed snifflet model, where the snifflet is also temporally dilated for each sniff based on inhalation duration. This is the model used in the main text. *Dilation: sniff dur*: the smoothed snifflet model, where the snifflet is temporally dilated for each sniff based on the total sniff duration. This has poorer predictive performance than the model in the main text. *Dilation: double*: the smoothed snifflet model, where the snifflet is temporally diluted at two intervals: sniff duration and the rest of the sniff. This model performed worse than pure inhalation duration dilation model.

Supplementary Figure 5

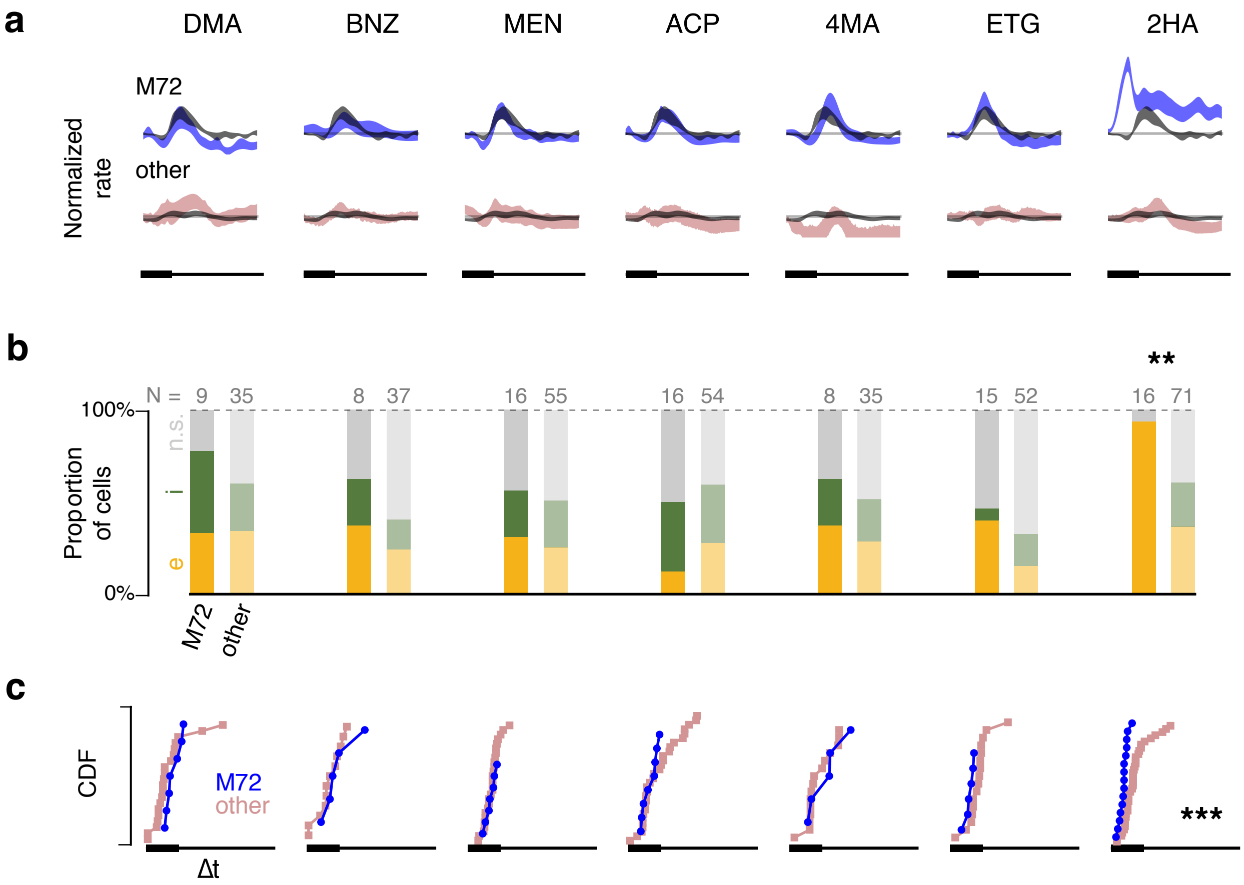

Same analyses and results as shown in Fig. 4, here obtained using the “no dilation” snifflet model (Supplementary Fig. 4, third column). The results are qualitatively identical to those shown in Fig. 4. Similar results for the experiment presented in Fig. 5 are obtained with this model as well (not shown). Panels displayed as in Figs. 4 & 5. **(a)** Mean snifflets of M72 (blue) and other (pink) MT cells, for each odor. **(b)** Distributions of the polarity of the first significant response of cells to each odor (e: excitatory; i: inhibitory; n.s.: not significant), compared between the M72 (bolded bars) and other (desaturated bars) MT sub-populations recorded. **(c)** Cumulative distributions of the latency of the first significant response, for M72 (blue) and other (pink) MT cells.

Supplementary Figure 6

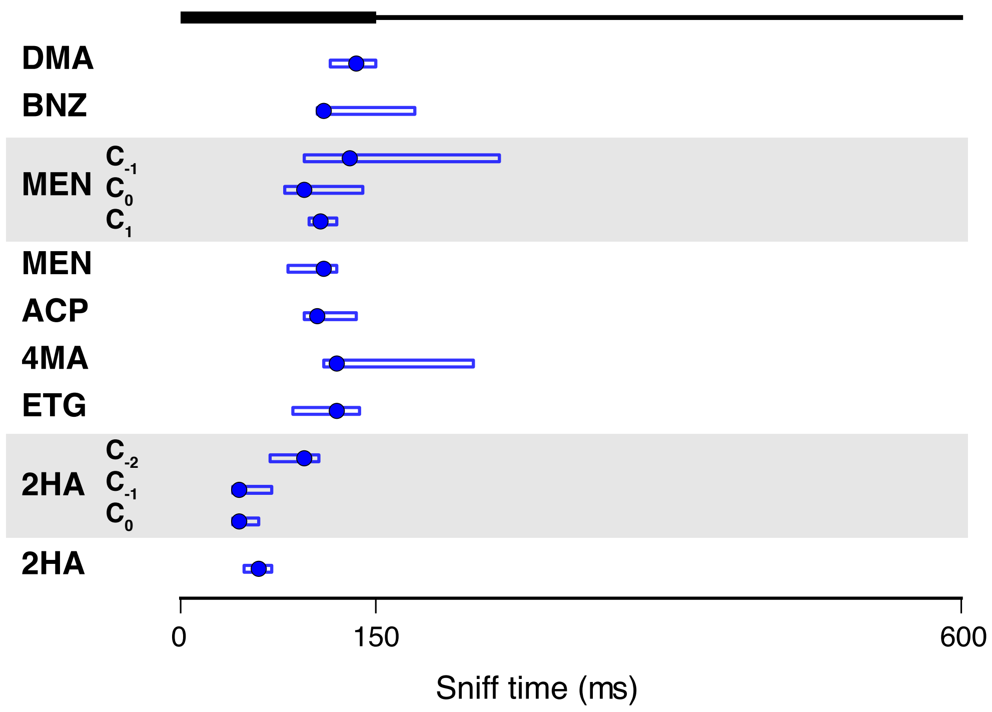

Average latency of the first significant response to each odor for M72-MT cells. Dots show the median first-response latency across cells; bars show the interquartile range. The odors against the white background summarize the latency distributions shown in Fig. 4c; the odors against the grey background summarize the latency distributions shown in Fig. 5c.

Supplementary Figure 7

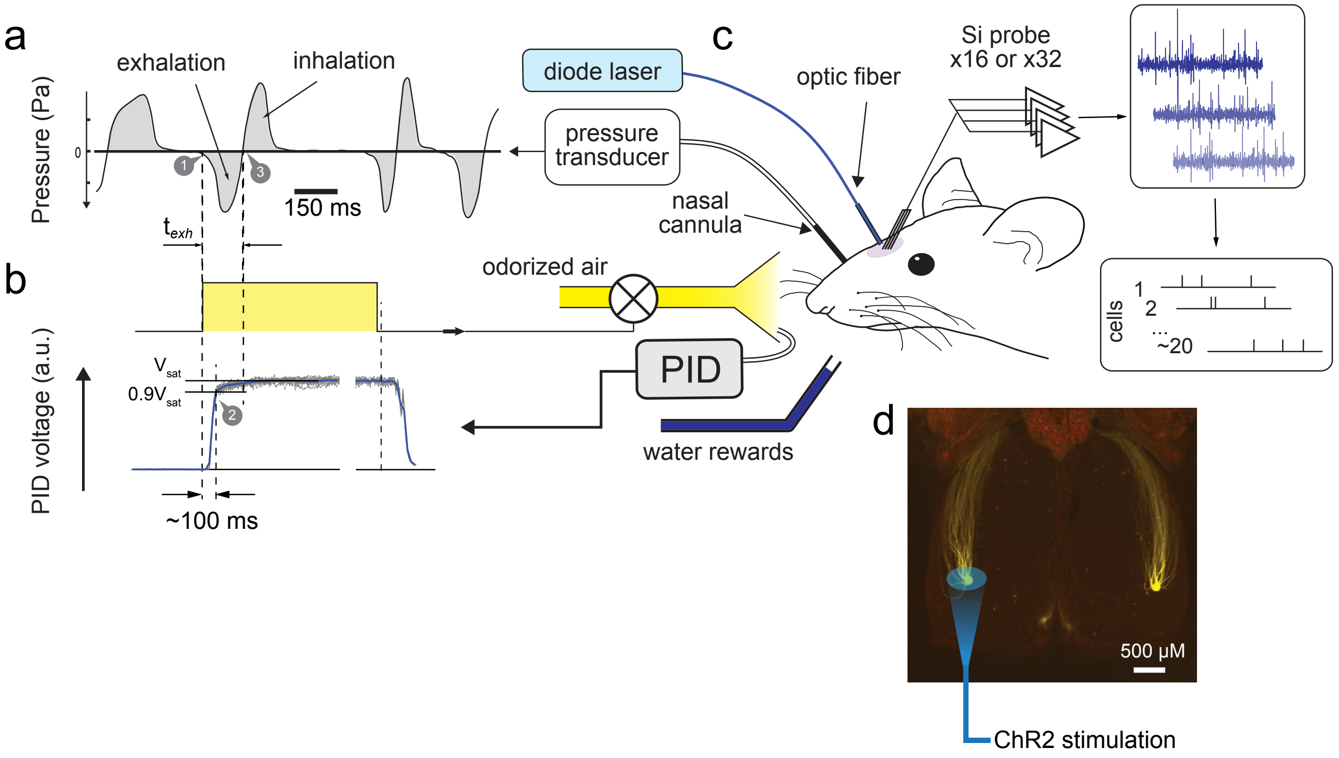

Electrophysiology experimental setup. Prior to the experiment, the mouse is implanted with a nasal cannula for respiratory pressure measurement and a head bar for head fixation (not shown). After recovery, the mouse is acclimated to the experimental apparatus. **(c)** At the beginning of the electrophysiological recording session, the animal is anesthetized with isofluorane. The YFP-expressing M72 glomerulus is located and the optical fiber placed above. A small craniotomy is made next to the glomerulus and a 16 or 32 site Si-probe is inserted, targeting the mitral cell layer near the glomerulus. The electrode is connected to the data acquisition system via a 32 channel headstage. Raw data is stored in the computer and spikes are sorted offline. All electrophysiological recordings are done after full recovery from anesthesia. **(a)** A typical pressure waveform from the nasal cavity is shown. **(b)** During the experimental session, a final valve, which delivers odors, is triggered at the onset of exhalation (**a**, time point 1). Such triggering ensures that when a mouse starts inhaling (time point 3), the odor concentration has reached a steady state (time point 2). The odor delivery system is calibrated prior to every experimental session using photoionization detector (PID). The typical profiles of PID traces for repeatable odor delivery at a given concentration are shown in (b): thin gray lines are individual trials, and blue line is an average profile. During the first 100 ms from the final valve trigger, odor concentration reaches 90% of its asymptotic value. For a majority of trials, exhalation duration (t_{exh}) is longer than 100 ms. **(d)** Dorsal view of olfactory bulbs of M72-ChR2-YFP mouse. Axons of M72 receptor neurons, co-expressing ChR2 and YFP converge to dorsal glomeruli in the left and right bulbs. Optical fiber connected to blue laser is positioned above dorsal M72 glomerulus.
